# High-risk alcohol use and anxiety and depression symptoms in adolescents and adults with type 1 diabetes mellitus: a cross-sectional study

**DOI:** 10.1186/s13098-015-0020-9

**Published:** 2015-03-24

**Authors:** Maria Aparecida Knychala, Maria Luiza Mendonça Pereira Jorge, Cinara Knychala Muniz, Priscila Neves Faria, Paulo Tannús Jorge

**Affiliations:** Graduate Program in Health Sciences, School of Medicine, Federal University of Uberlândia, Uberlândia, MG CEP: 38400-902 Brazil; Management of Nutrition and Dietetics, Clinical Hospital, Federal University of Uberlândia, Uberlândia, MG Brazil; Mathematics Faculty, Federal University of Uberlândia, Uberlândia, MG Brazil

**Keywords:** Type 1 diabetes mellitus, Prevalence, Alcohol, Anxiety, Depression, Glycemic control

## Abstract

**Background:**

The medical literature shows that alcohol consumption is common among diabetic individuals and is associated with poor adherence to treatment, resulting in increased morbidity and mortality. However, no study has assessed the association between high-risk alcohol consumption and the presence of anxiety and depression in individuals with type 1 diabetes mellitus (1DM).

**Methods:**

The present cross-sectional study was conducted in Uberlândia, Brazil, and it assessed 209 outpatients in relation to alcohol consumption and the presence of anxiety and depression symptoms, using the Alcohol Use Disorders Identification Test (AUDIT), the Hospital Anxiety and Depression (HAD) scale, and glycemic control, according to the levels of glycated hemoglobin (HbA1c). The chi-square test and logistic regression analysis were used to investigate the association between the investigated variables.

**Results:**

The prevalence of high-risk alcohol consumption (AUDIT ≥ 8) among individuals with 1DM was high, specifically 24.9% among the entire group of subjects, 12.9% among the adolescents, 14.7% among the females, and 34.6% among the males. In comparisons based on gender and age, the odds of high-risk drinking were higher among males and participants aged 30 to 40 years (33.9%). The frequency of high-risk alcohol consumption did not differ as a function of gender among adolescents (females: 9.0%, males: 16.2%; p = 0.374). There was a linear trend in proportions related to the scores of anxiety and depression symptoms with high-risk alcohol consumption scores, indicating the association of these variables (p = 0.0229 and p = 0.0262, respectively). Moreover, the odds of female subjects exhibiting anxiety and depression symptoms were higher (odds ratio – OR: 4.4 and OR: 7.4, respectively). Glycemic control did not exhibit an association between high-risk alcohol consumption and the presence of anxiety and depression symptoms.

**Conclusions:**

The frequency of high-risk drinking increased along with age and was greater among males; however, this frequency did not exhibit differences in terms of gender among adolescents. There was a positive association between high risk alcohol consumption and anxiety and depression symptoms, although glycemic control was inadequate in most of the sample independent of alcohol consumption and the presence of anxiety and depression symptoms.

## Background

The number of individuals with diabetes mellitus increases every year and is estimated to reach 592 million by 2035, with a faster increase expected in poor and developing countries [1]. In Brazil, the number of individuals with diabetes mellitus is estimated at 11.9 million, 5 to 10% of which are type 1 diabetes mellitus (1DM) [1]. Studies conducted in different parts of the world also show that the incidence of 1DM among children and adolescents is increasing [2].

The literature shows that the age at which alcohol consumption begins is falling steadily among adolescents and young adults, and therefore the risks associated with alcohol abuse are affecting a younger population [3,4]. Studies conducted in Brazil have shown a high prevalence of frequent alcohol use (one to four times per week) among adolescents (8%) and adults (24%) [5,6]. However, there are few studies on the frequency of alcohol consumption by individuals with 1DM [7,8] as a whole, and no studies have been conducted in Brazil.

Review studies indicate that the alcohol use is common among individuals with diabetes mellitus, and this behavior is associated with poor adherence to treatment. This association may result in negative outcomes [9], including increased morbidity [10] and up to twofold increases in mortality [11], besides being a risk factor for trauma due to external causes [12-14].

The World Health Organization (WHO) recommends the Alcohol Use Disorders Identification Test (AUDIT) as a screening instrument [15] to allow early and effective treatment.

Several studies have found that the prevalence of depression is two to three times higher among individuals with diabetes mellitus compared to the overall population; in regard to gender, this prevalence is also higher among females than males. Although depression occurs up to three times more frequently in adolescents with 1DM, the causes of this association remain unknown [16,17]. Anxiety and depression may hinder proper glycemic control, as one study found that individuals with a positive screen for mental diseases had twice the odds of having poor glycemic control compared to those without them [18]. There are several factors such as age at diagnosis, diabetes mellitus duration and the type of treatment that influence the glycemic control [19,20].

The aim of the present study was to investigate the degree of alcohol consumption, the prevalence of anxiety and depression symptoms, and the level of glycemic control of adolescents and adults with 1DM who were followed by an endocrinology outpatient clinic.

## Methods

The present cross-sectional study was conducted at the outpatient clinics of the Federal University of Uberlândia and the Municipal Center for Diabetics’ Care of Uberlândia. These centers are located in Southeastern Brazil, and participants were interviewed from November 2011 to October 2012. This study used a convenience sample and was approved by the research ethics committee of the Federal University of Uberlândia (no. 251/2011) and the Municipal Health Secretary of Uberlândia. Diagnosis of 1DM was established based on the criteria formulated by the American Diabetes Association (ADA) [21]. The data were collected by one of the investigators during individual interviews conducted in a private room. During these interviews, demographic data were collected using a form, the AUDIT instruments and the Hospital Anxiety and Depression (HAD) scale were applied. All of the participants, or their parents or guardians in case individuals were younger than 18 years, signed an informed consent form. In addition, adolescents signed an information letter, as requested by the research ethics committee of the Federal University of Uberlândia. The inclusion criteria consisted of a diagnosis of 1DM according to ADA criteria [21], age of 14 to 40 years, and consent to participate. The age of 14 was set as the minimum, based on the study that validated the HAD in Brazil [22,23]. The exclusion criteria were schizophrenia; bipolar disorder; heart, liver, or kidney failure; cognitive deficit; or other diseases that could impair the data interpretation.

### Characteristics of the case series

The following data were recorded: age (14–19, 20–29, and 30–40), gender, marital status, educational level, current smoker, use of illegal drugs, presence of comorbidities, and family history of alcohol use. As all the patients are 1DM they were using insulin. The subject’s educational level was categorized as less than 8 years or more than 8 years of formal schooling. In regard to the participants’ marital status, we considered subjects without a partner as single and those legally married or in stable unions as married.

### Instrument to assess the degree of alcohol use

AUDIT was used to assess alcohol use; this scale was formulated by the WHO [15] and translated and validated in Brazil [24] and in adolescents [25-27]. AUDIT is an international instrument that was developed to screen high-risk drinking based on the measurement of alcohol consumption, personal and social consequences, and alcohol dependence in the past 12 months. Based on their total scores, the participants were classified in one of four possible risk categories: scores up to 7 indicated low-risk drinking; 8 to 15, hazardous drinking; 16 to 19, harmful drinking; and over 20, possible alcohol dependence [15]. On these grounds, a score of 8 points was selected as the cutoff value for defining high-risk alcohol use, as this value was shown to exhibit 100% sensitivity and 76% specificity [24], as well as respective values of 82% and 72% in the case of adolescents [27]. Individuals who reported no alcohol consumption in the past year were considered abstemious [28].

### Instrument to assess anxiety and depression symptoms - HAD

HAD comprises 14 items: 7 assessing anxiety (HAD-A) and 7 assessing depression (HAD-D). Each item can be scored from 0 to 3, and the highest total score in each subscale is thus 21. The cutoff point selected in the present study to define the frequency of anxiety and depression symptoms as positive was a score of 8 points, as this value exhibited satisfactory sensitivity and specificity in both the anxiety (74% and 74.2%, respectively) and the depression (85.7% and 72.4%, respectively) subscales in the validation study conducted in Brazil [22].

### Assessment of glycemic control

The data related to glycated hemoglobin (HbA1c) values during the study period were collected from the participants’ clinical records. HbA1c was measured by means of the immunoturbidimetric method (kits by laboratories ABBOT and Roche, and Architect C 8000 and Cobas 6.000 equipment; reference value: 4.0% to 6.0%). Values lower than 7.0% in adults and lower than 7.5% in adolescents represented good glycemic control; values ranging from 7.0 to 9.0% in adults and from 7.5 to 9.0% in adolescents represented moderate glycemic control; and values over 9.0% were considered poor glycemic control [29].

### Statistical analysis

The association between qualitative dichotomous variables was assessed by means of the two binomial proportions or the chi-square (*χ*^2^) test, and those that exhibited statistical significance were subjected to multiple logistic regression analysis, in which alcohol consumption was the dependent variable; these results were expressed as the odds ratio (OR). In the chi-square (*χ*^2^) test continuity correction was used whenever the expected frequency was less than 5. Nominal variables with more than two categories were analyzed by means of multiple comparison tests for proportions.

It was used the Cochran-Armitage trend test, sometimes also referred to as the trend on proportions test, allows to test if a series of proportions (or the corresponding counts, stored in Rx2 contingency table), can be considered as varying linearly with an ordinal or continuous score variable. It is related to the Chi-square independence test that allows to test if there is relationship between the rows and the columns of a contingency table. The Cochran-Armitage test allows you to take into account a ranking among the rows, based on scores [30]. Statistical analyses were performed using BioEstat 5.0 software [31] and R 2.6.1 for Windows® [32] and JMP® 2000 [33]. The significance level was set at 5.0%.

## Results

The group of individuals with 1DM treated at the outpatient clinics of the Federal University of Uberlândia and the Municipal Center for Diabetics’ Care within the investigated age range included 427 patients. A total of 221 consecutive patients were invited to participate in the study, although 6 (2.7%) refused to participate, and 6 were excluded, including 4 (1.8%) due to cognitive deficit and 2 (0.9%) to kidney failure. Approximately 97.0% of the initial sample agreed to participate, and 94.6% remained in the study after application of the exclusion criteria. The final sample thus comprised 209 individuals with 1DM. The median age of the sample was 23 years old.

Males constituted 51.0% of the sample, and there was no significant gender difference (p = 0.6248). In addition, 69.0% of the subjects were single, and 88.0% had attended formal schooling for at least 8 years (Table 1).

**Table 1:** **Socio- demographic characteristics of individuals with type 1 diabetes**

**Variables**	**Female (n = 102)**		**Male (n = 107)**		**p-value**	**Total (n = 209)**	
	**n**	**%**	**n**	**%**		**n**	**%**
**Age range (n = 209)**							
**14 [−] 19 years old**	33	32.3	37	34.6	0,733	70	33.5
**20 [−] 29 years old**	36	35.3	47	43.9	0,202	83	39.7
**30 [−] 40 years old**	33	32.3	23	21.5	0,076	56	26.8
**Marital status**							
**Married**	38	37.2	27	25.2	0,061	65	31.1
**Single**	64	62.7	80	74.8	0,061	144	68.9
**Educational level**							
**<8 years**	12	11.8	14	13.1	0,772	26	12.4
**≥8 years**	90	88.2	93	86.9	0,772	183	87.6

Approximately 9.0% of the participants were current smokers, and 12.0% used illegal drugs. In approximately 64.0% of subjects, the time elapsed since the diagnosis of 1DM was longer than 5 years. The level of HbA1c was very high (≥9.0%) in most of the participants (52.6%), and 72.2% showed a positive family history of alcohol consumption (Table 2).

**Table 2:** **Clinical characteristics of the participants**

**Variables**	**Female (n = 102)**		**Male (n = 107)**		**Total (n = 209)**	
	**n**	**%**	**n**	**%**	**n**	**%**
**High-risk alcohol use AUDIT ≥8**						
Yes	15	14.7	37	34.6	52	24.9
No	87	85.3	70	65.4	157	75.1
**Anxiety symptoms HAD-A ≥8**						
Yes	38	37.2	24	22.4	62	29.7
No	64	62.7	83	77.6	147	70.3
**Depression symptoms HAD-D ≥8**						
Yes	18	17.6	5	4.7	23	11.0
No	84	82.3	102	95.3	186	89.0
**Current smoker**						
Yes	05	04.9	14	13.1	19	9.1
No	97	95.1	93	86.9	190	90.9
**Drug use**						
Yes	04	3.9	21	19.6	25	12.0
No	98	96.1	86	80.4	184	88.0
**Time since diagnosis of 1DM**						
≤5 years	35	34.3	41	38.3	76	36.4
>5 years	67	65.7	66	61.7	133	63.6
**Parental alcohol use**						
Yes	76	74.5	75	70.1	151	72.2
No	26	25.5	32	29.9	58	27.7
**HbA1c**						
Good control	7	6.9	12	11.2	19	9.1
Moderate control	39	38.2	41	38.3	80	38.3
Poor control	56	54.9	54	50.5	110	52.6

AUDIT: *Alcohol Use Disorders Identification Test*; HAD-A: *Hospital Anxiety and Depression*– anxiety subscale; HAD-D: *Hospital Anxiety and Depression*– depression subscale.

High-risk alcohol use was greater among males aged 30 to 40, although this variable did not exhibit a significant difference between genders among adolescents (p = 0.374) (Table 3).

**Table 3:** **Comparison of high-risk alcohol use (AUDIT ≥8) between genders per age range**

	**Female**		**Male**		
**Age range years (n = 209)**	**AUDIT (n = 15) Female (n = 102)**	**%**	**AUDIT (n = 37) Male (n = 107)**	**%**	**p-value**
14[−]19 (n = 70)	3/33	9.1	6/37	16.2	0.374
20[−]29 (n = 83)	6/36	16.7	18/47	38.3	0.031
30[−]40 (n = 56)	6/33	18.2	13/23	56.5	0.0029

AUDIT: *Alcohol Use Disorders Identification Test*; p ≤ 0.05.

Table 4 shows that the prevalence of high-risk alcohol use (AUDIT ≥8) was greater among males, the oldest age range, users of illegal drugs, current smokers, and participants with a positive family history of alcohol consumption.

**Table 4:** **Prevalence of high-risk alcohol use according to the variables examined**

**Variables**	**High-risk alcohol use (AUDIT ≥ 8)**							
	**AUDIT (n = 52) Total (n = 209)**	**%**	**Crude OR**	**95%CI**	**p-value**	**Adjusted OR**	**95%CI**	**p-value**
**Gender**								
Female	15/102	14.7	1					
Male	37/107	34.6	3.1	1.5-6.0	0.0009	2.8	1.3-6.2	0.0108
**Age range (years)**								
14[−]19	9/70	12.9	1		a#			
20[−]29	24/83	28.9	2.8	1.2-6.4	ab#			
30[−]40	19/56	33.9	3.5	1.4-8.5	b#	1.1	1.0-1.1	0.0135
**FH (alcohol use)**								
No	6/58	10.3	1					
Yes	46/151	30.5	3.8	1.5-9.5	0.00259	7.0	2.2-2.5	0.0010
**Drug use**								
No	35/184	19.0	1					
Yes	17/25	68.0	9.0	3.6-22.6	<0.0001	5.9	1.9-17.9	0.0017
**Current smoker**								
No	38/190	20.0	1					
Yes	14/19	73.7	11.2	3.8-33.0	<0.0001	8.2	2.2-30.6	0.0016
**HbA1c**								
Good control	4/19	21.0	1					
Moderate control	22/80	27.5	1.4	0.4-4.8				
Poor control	26/110	23.6	1.2	0.3-3.8	0.7658	NS		

AUDIT: *Alcohol Use Disorders Identification Test*; FH: family history; p ≤ 0.05; # proportions followed by the same letter do not differ. HbA1c: glycated hemoglobin; OR: *Odds Ratio*; CI: confidence interval.

Among the participants classified as high-risk drinkers, 29.0% (IC 95%: 0.7-2.6) exhibited anxiety symptoms and 34.8% (IC 95%: 0.7-4.3) depression symptoms. There was a linear trend in proportions related to the scores of anxiety and depression symptoms with high-risk alcohol consumption scores, indicating the association of these variables (p = 0.0229 and p = 0.0262, respectively), data not shown in table.

Table 5 shows that the odds of exhibiting anxiety symptoms were 2.0 times higher among females compared to males (IC 95%: 1.1-3.8), while no difference was found when age was considered. The odds of drug users exhibiting anxiety symptoms were also 2.5 times higher compared to non-users (IC 95%: 1.1-5.8). Moreover, the presence of anxiety symptoms exhibited an association with the presence of depressive symptoms but not with glycemic control (Table 5).

**Table 5:** **Prevalence of anxiety symptoms according to the variables examined**

**Variables**	**Anxiety symptoms – HAD-A ≥8**				
	**Anxiety (n = 62)/Total (n = 209)**	**%**	**OR**	**95% CI**	**p-value**
**Gender**					
Male	24/107	22.4	1		
Female	38/102	37.2	2.0	1.1-3.8	0.019
**Age range (years)**					
14[−]19	17/70	24.3	1		a#
20[−]29	21/83	25.3	1.1	0.5-2.2	a#
30[−]40	24/56	42.7	2.3	1.1-5.0	a#
**Drug use**					
No	50/184	27.2	1		
Yes	12/25	48.0	2.5	1.1-5.8	0.0324
**HAD-D**					
No depression	43/186	23.1	1		
Depression	19/23	82.6	15.8	2.4-11.0	<0.0001
**HbA1c**					
Good control	3/19	15.8	1		a#
Moderate control	23/80	28.7	2.1	0.6-8.1	a#
Poor control	36/110	32.7	2.6	0.7-9.5	a#

HAD-A: *Hospital Anxiety and Depression* – anxiety subscale; p ≤ 0.05; # proportions followed by the same letter do not differ. HbA1c: glycated hemoglobin; OR: *odds ratio*; CI: confidence interval.

The odds of women exhibiting symptoms of depression were 4.4 higher compared to men (IC 95%: 1.6-12.3), and the odds of adults exhibiting such symptoms were 7.4 higher compared to the other age ranges (IC 95%: 2.0-27.5). In addition, smoking increased the risk of depression by 6.3-fold (IC 95%: 2.2-18.4) (Table 6).

**Table 6:** **Prevalence of depression symptoms according to the variables examined**

**Variables**	**Depression symptoms – HAD-D ≥8**				
	**Depression (n = 23)/Total (n = 209)**	**%**	**OR**	**95% CI**	**p-value**
**Gender**					
Male	5/107	4.7	1		
Female	18/102	17.6	4.4	1.6-12.3	0.0027
**Age range (years)**					
14[−]19	3/70	4.3	1		a#
20[−]29	6/83	7.2	1.7	0.4-7.2	a#
30[−]40	14/56	25.0	7.4	2.0-27.5	b#
**Current smoker**					
No	16/190	8.4	1		
Yes	7/19	30.8	6.3	2.2-18.4	0.00016
**HbA1c**					
Good control	1/19	5.3	1		a#
Moderate control	8/80	10.0	2.0	0.2-17.0	a#
Poor control	14/110	12.7	2.6	0.3-21.2	a#

HAD-D: Hospital Anxiety and Depression – depression subscale; p ≤ 0.05; # proportions followed by the same letter do not differ. HbA1c: glycated hemoglobin; OR: *odds ratio*; CI: confidence interval.

## Discussion

The present study found high prevalence of high-risk drinking among adolescents and adults with 1DM. In particular, this prevalence was greater in males and older participants but there was no gender difference among adolescents. This present study results concerning the prevalence of high-risk drinking among individuals with diabetes mellitus are similar to those previously reported for the overall Brazilian [34-36] and the United States [37,38] population.

Although no previous studies have assessed the prevalence of alcohol use among individuals with 1DM in Brazil, studies conducted in Chile and Ireland found prevalence rates varying from 7% to 30.1% [7,8]. Such wide variation is likely due to the use of different tools and terminologies used to diagnose alcohol consumption, as well as differences in the population age assessed and the study setting.

In our study, in contrast to females, the proportion of abstemious individuals was greater among males (22.4%) compared to the overall population (8.7%) reported by Silveira [28] in a study conducted in Brazil with 1,464 participants. According to the literature, alcohol consumption is greater among males, although we observed similarities in this prevalence between genders among adolescences, which corroborates previous findings [28,39].

Our results revealed that alcohol consumption increased with age. This finding agrees with the results of a study conducted in Chile, which found that the rate of alcohol consumption by older adolescents was higher than that observed in the younger [8]. Other authors have reported that the onset of alcohol use at the beginning of adolescence is associated with a greater frequency of abuse and future dependence [40].

Self-care is a fundamental factor in the treatment of diabetes mellitus, and alcohol consumption might be considered a marker of risk for non-adherence to treatment, which results in patients being less prone to seek routine care [9]. The rate of anxiety symptoms found in the present study (29.7%) is higher than that reported for the overall population in a recent study (19.9%) [41] as well as that reported by a study conducted on diabetic individuals (21.3%) [18]; however, our rate of anxiety symptoms was similar to that reported by another study also conducted with diabetic individuals (32%) indicating that high risk drinkers are more prone to anxiety and depression symptoms [7]. The frequency of anxiety and depression symptoms was also higher among females, which agrees with the results of studies conducted on diabetic individuals [7,42] or the overall population [43]. In addition, the frequency of depression symptoms we found was similar to that reported for the overall population [41] and diabetic individuals [18].

The association between anxiety and depression symptoms observed in our sample of individuals with diabetes is in agreement with previous reports in the literature. Moreover, this association has been correlated with lower rates of adherence to treatment, greater severity of depression, and eventually with greater mortality [44,45].

In more than half of the participants in our study, the HbA1c level was ≥9%, which is indicative of poor control of diabetes mellitus, and this finding agrees with the results reported by other authors [46,47]. However, different from other studies [48], we did not detect an association between glycemic control and high-risk alcohol use. The reason for this observation was most likely because of the small number of participants who exhibited good glycemic control (the HbA1c level was <7% in only 9% of the sample). This finding may be explained by the eventual presence of other factors that exert an influence on glycemic control, such as the length of diabetes mellitus, age, use of medication, adherence to treatment, family conflict, anxiety, and depression [47,49-51]. Moreover, glycemic control did not exhibit an association with the presence of anxiety symptoms, thus disagreeing with the findings of other authors [7,52].

The results of our logistic regression analysis agree with those studies that emphasized the wide variety of variables that might influence alcohol consumption, including family and environmental factors, depression, anxiety, smoking, and the use of illegal drugs [40,53-61].

The proportion of current smokers in the present study was lower than that previously reported by studies conducted in the overall population [62] and diabetic individuals [8]. Nevertheless, this prevalence remains far from the desired level, as no diabetic individual should smoke because tobacco use is associated with a substantial increase in the complications of this disease, particularly those related to the cardiovascular, renal and neurological systems [29].

The frequency of a positive family history of alcohol consumption was high in the assessed sample, which suggests that the influence exerted by the family relative to high-risk drinking is strong. Living with both parents and sharing at least one meal with parents or guardians most days of the week have been shown to protect against high-risk drinking; in addition, accurate knowledge of what adolescents do in their free time has a protective effect against the risk of smoking and the use of alcohol and drugs [40,53]. The frequency of the use of illegal drugs was high in the present sample, which agrees with the result reported by a review study conducted on the overall population of the United States [63].

### Clinical implications of the study

Patients with diabetes mellitus who are high-risk drinkers exhibit more frequent and severe health problems. The American Academy of Pediatrics recommends investigating depression and alcohol use in all adolescents, and the ADA emphasizes the specific relevance of such assessment for the diabetic population [29,37,64]. Our study stresses the need for healthcare professionals to identify high-risk alcohol consumption and to promote a type of follow up that ensures successful glycemic control.

### Study strengths

Validated screening tests are useful for the identification of patients who might benefit from early diagnosis and treatment and for diabetic individuals who are at high risk for alcohol consumption and the presence of comorbidities, such as anxiety and depression. Further studies with larger samples and in other geographical areas are needed to establish the reproducibility of our results. Longitudinal studies are indispensable to investigate the causal relations among these factors, as well as to assess the effectiveness of interventions aiming at improving glycemic control in this population.

### Study limitations

The present study exhibited limitations inherent to cross-sectional studies, in which causal factors and directions cannot be assessed. Another limitation derived from the fact that the present study was conducted at a reference center and used a convenience sample. For these reasons, the data could not be extended to other settings, such as primary care, or to other geographical areas.

## Conclusions

The prevalence of high-risk alcohol consumption and symptoms of anxiety and depression was high in the assessed sample of adolescents and adults with 1DM. The frequency of high-risk drinking increased with age, and its prevalence was greater among males; however, differences in prevalence were not significant between genders in adolescents. There was a positive association between high risk alcohol consumption and anxiety and depression symptoms, although glycemic control was inadequate in most of the sample independent of alcohol consumption and the presence of anxiety and depression symptoms.

## Abbreviations

ADA: American diabetes association
AUDIT: Alcohol use disorders identification test
DM: Diabetes mellitus
1DM: Type 1 diabetes mellitus
USA: United States of America
HAD: Hospital anxiety and depression
HbA1c: Glycated hemoglobin
CI 95%: Confidence interval 95%
OR: Odds ratio
WHO: World Health Organization
